# Deep Femoral Artery Identification Using Contrast-Enhanced Computed Tomography Images for the Verification of Vascular Injury Risks in Hip Surgery

**DOI:** 10.7759/cureus.89803

**Published:** 2025-08-11

**Authors:** Yuta Hieda, Hyonmin Choe, Hiroyuki Ike, Koki Abe, Masashi Shimoda, Ken Kumagai, Naomi Kobayashi, Yutaka Inaba

**Affiliations:** 1 Department of Orthopaedic Surgery, Yokohama City University, Yokohama, JPN; 2 Department of Orthopaedic Surgery, Yokohama City University Medical Center, Yokohama, JPN

**Keywords:** contrast-enhanced computed tomography images, deep femoral artery, femoral nailing, total hip arthroplasty (tha), vascular injury

## Abstract

Iatrogenic deep femoral artery (DFA) injury is a serious complication of hip surgery, often resulting from screw or wire placement in the femur owing to the limited visibility of the DFA and its branches during femoral penetration. We aimed to identify the course and location of DFA perforating branches using an imaging-based approach to improve surgical planning and prevent vascular injury during hip procedures, which has not been thoroughly evaluated in prior anatomical studies. We consecutively enrolled 20 female and 20 male participants with unilateral hip osteoarthritis. Contrast-enhanced computed tomography images of the unaffected side were used to identify the DFA. Associations between participant demographics and DFA branch location and trajectory were analyzed. The distance from the apex of the greater trochanter (GTR) to the first DFA perforating branch was significantly shorter in females than in males (mean: 100 (range, 77-122) vs. 113 (range, 99-131) mm, P < 0.001), whereas no significant difference was found for the second branch (mean: 154 (range, 108-242) vs. 160 (range, 128-235) mm, P = 0.73]. The DFA ran within 5 mm of the femur on the medial-posterior aspect at 140 mm and 200 mm distal to the apex of the greater trochanter, typical insertion sites for distal cortical screws in intramedullary nailing for hip fractures. In females, the first DFA perforating branch occurs more proximally than in males, necessitating caution during femoral wiring at this level. These findings help refine anatomical understanding of the DFA course, supporting safer surgical planning for intramedullary fixation. The small sample size (n = 40) is one of the limitations and may affect generalizability.

## Introduction

The deep femoral artery (DFA) typically branches from the common femoral artery approximately 40 mm distal to the inguinal ligament [1]. It runs posteriorly between the adductor longus and adductor brevis muscles, giving rise to multiple perforating branches that supply the posterior and lateral compartments of the thigh [2]. Intraoperative injury to the DFA is a serious complication of total hip arthroplasty (THA) or revision THA for osteoarthritis or intramedullary femoral nailing for proximal femoral fractures [3-7]. DFA has been reported to give rise to multiple branches that perforate the femur while the main trunk runs alongside it [8]. These perforating branches typically course along the medial-posterior side of the femur and usually include 3-4 branches [8]. Iatrogenic DFA injury may occur when the femur is wired or screwed during hip surgery, as these procedures involve blind manipulation along the femur’s medial aspect [4,7,9]. Although several studies have described the anatomical location of the DFA [10-12], these were primarily cadaveric or based on stratified imaging levels. The precise course, angulation, and branch penetration sites relative to surgical landmarks remain inadequately characterized, particularly in the context of modern surgical procedures such as cerclage wiring and intramedullary nailing.

In THA and intramedullary femoral nailing, cerclage wiring around the lesser trochanter (LTR) or femoral shaft is often used to stabilize bone fragments [13-15]. To prevent vascular injury during wiring, the surrounding soft tissue must be adequately protected. If wiring is performed at a site where the DFA branches perforate the femur, arterial injury may be unavoidable. Damage to the first or second perforating branch, which tends to be larger, may cause substantial perioperative blood loss. Therefore, identifying safe zones for femoral wiring is clinically important.

Intramedullary nailing also requires fixation with distal cortical screws, typically placed at approximately 140 mm from the device head with short nails and approximately 200 mm with semi-long nails [16]. Recently, cases of postoperative anemia caused by DFA injury due to blind drilling or fluoroscopy-guided screw insertion have been reported [3-5]. Therefore, understanding the precise location and angle of the DFA in regions where screws are inserted is critical. Although previous studies have demonstrated that the DFA runs along the medial-posterior aspect of the femur based on stratified level analyses [10,17], they have not focused on the specific heights where distal screws are inserted or on the DFA’s location within actual surgical fields.

This study aimed to evaluate the course of the DFA and its perforating branches at key surgical landmarks. Specifically, we examined the first and second perforating branches, the LTR, and positions at heights of 140 and 200 mm from the apex of the greater trochanter (GTR) and compared findings between male and female participants.

## Materials and methods

Study design and population

This retrospective study enrolled 40 participants with unilateral hip osteoarthritis, including 20 consecutive females who underwent unilateral THA between December 2020 and March 2021 and 20 consecutive males between December 2019 and March 2021. The mean age of the participants was 66.3 ± 10.6 years (range, 48-88). All participants were ethnically Japanese and underwent contrast-enhanced computed tomography (CT) within one week after surgery. Exclusion criteria included absence of a postoperative contrast-enhanced CT scan (females: n = 5, males: n = 2), femoral head deformity such as osteoarthritis (females: n = 10, males: n = 4), history of leg fracture or osteomyelitis (females: n = 0, males: n = 1), and inability to detect DFA course owing to metal artifacts (females: n = 0, males: n = 0). The DFA on the non-affected side was analyzed in all included participants. The study was approved by the Institutional Review Board of Yokohama City University (approval no. F241205074). Informed consent was obtained through an opt-out process on the hospital website.

Measurements

Preoperative age, sex, height, body mass index (BMI), and femoral rotation were recorded. CT scanning (Sensation16; Siemens AG, Erlangen, Germany; 120 kV, 300 mA, 1.5-mm slice thickness) was performed from the first lumbar vertebra to the distal femur. The first and second branches of the DFA were identified on contrast-enhanced CT images of the unaffected side. All measurements were performed by an orthopaedic surgeon with experience in musculoskeletal imaging.

**Figure 1:**
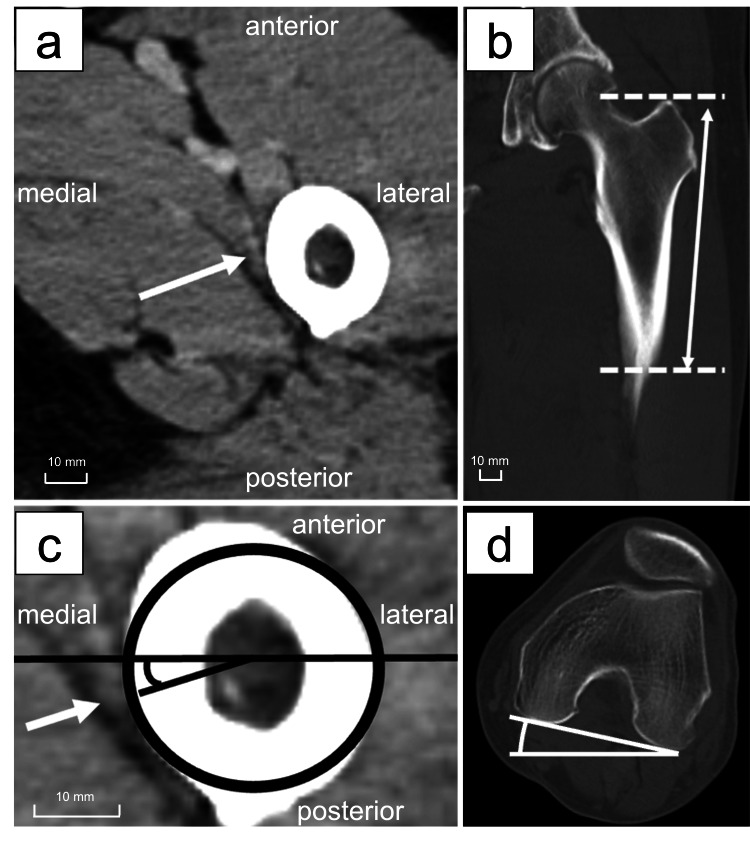
Location of the deep femoral artery (DFA) branch and measurement of the femoral rotation angle. Penetrating branches of the DFA are indicated by white arrows. The height of the DFA penetration site from the apex of the greater trochanter (GTR) is marked with a double white arrow. (a) The horizontal section image of DFA penetrating the femur. (b) The coronal section image measuring DFA height or the lesser trochanter from the apex of the greater trochanter. (c) The angle of DFA penetration into the femur. (d) The femoral rotation angle at the femoral condyle.

The GTR apex was defined as the most proximal level on axial CT images at which the greater trochanter was visible. The LTR was defined as the most distal level on axial CT images at which the prominence of the lesser trochanter disappeared. First, the perforating branches of the DFA were traced from proximal to distal in the axial plane (Fig. 1a). The distance from the apex of the GTR to the perforation sites of the first and second DFA branches was measured by drawing a straight line from the GTR apex to the level of the knee center in the coronal view (Fig. 1b). The insertion points of the DFA branches were further analyzed by calculating the angle between the posterior femoral condyle axis (PFCA) and the line connecting the femoral center point to the DFA insertion point (DFA axis) (Fig. 1c, 1d). The femoral center point was approximated as the center of a regular circle in the axial image.

**Figure 2:**
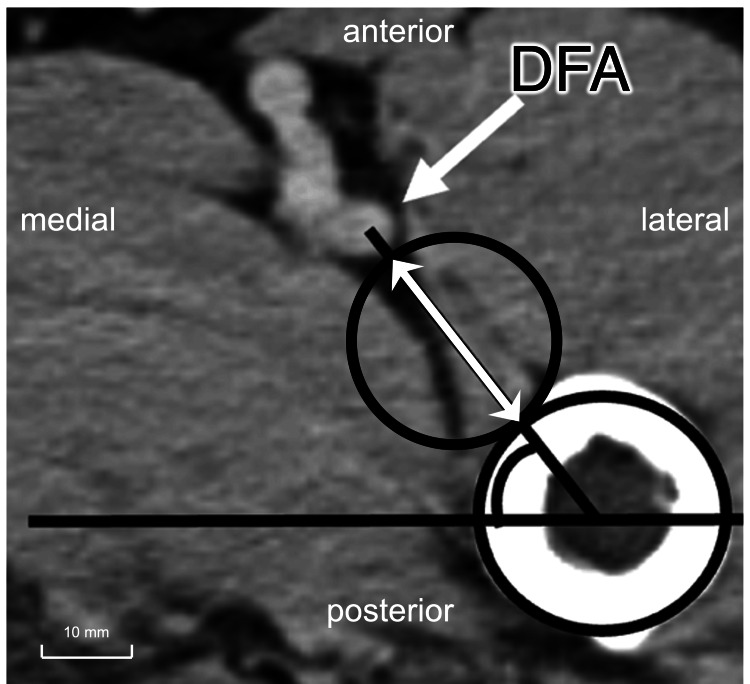
Assessment of the anatomical location of the deep femoral artery (DFA). The white arrows indicate the distance between the DFA and the femur.

Second, the distance and angle from the femoral cortex to the DFA were measured at three levels: the distal end of the LTR and at 140 and 200 mm distal to the GTR apex. The LTR level was defined as the slice in which the LTR was no longer visible in the axial view. The distance from the GTR apex to the distal end of the LTR was also recorded (Fig. 2). The 140- and 200-mm levels represented typical insertion sites for distal cortical screws in short and semi-long nails. At each level, the DFA position was assessed by measuring the distance from the femoral cortex to the artery and the angle between the PFCA and DFA.

Third, correlations between participant height and both DFA height and the shortest femur-to-DFA distance were evaluated.

Statistical analysis

All anatomical measurements were based on absolute values without adjustment for the participant's height or body size. The Wilcoxon signed-rank test was used to assess statistical significance for non-normally distributed data. Spearman’s correlation coefficient (R) was calculated to examine associations between height and arterial measurements. The strength of the correlation was interpreted based on commonly accepted thresholds: |R| ≥ 0.10 as small, ≥ 0.30 as moderate, and ≥ 0.50 as large effect sizes. Analyses were performed using JMP Pro version 17.0 (SAS Institute, Inc., Cary, NC, USA). A P-value of <0.05 was considered statistically significant.

## Results

Demographic data

Age and BMI did not differ significantly between males and females (Table 1). However, males were significantly taller than females (1.67 ± 0.04 vs. 1.54 ± 0.30 m, P < 0.0001). The femoral external rotation angle was significantly smaller in females than in males (10.5° ± 13.4° vs. -2.1° ± 12.4°, P = 0.006).

**Table 1:** Demographic data Data are presented as mean ± standard deviation. BMI, body mass index; CI, confidence interval *Wilcoxon signed-rank test. **Femoral rotation is expressed as a positive angle for external rotation.

	Female (n = 20)	Male (n = 20)	P-value*
Age (years)	65.4 ± 11.0 (95% CI: 60.2–70.6) (n = 20)	67.2 ± 10.3 (95% CI: 62.3–72.0) (n = 20)	0.481
Height (m)	1.54 ± 0.30 (95% CI: 1.49–1.56) (n = 20)	1.67 ± 0.04 (95% CI: 1.66–1.69) (n = 20)	<0.001
BMI (kg/m^2^)	24.6 ± 4.44 (95% CI: 22.5–26.7) (n = 20)	25.3 ± 3.72 (95% CI: 23.5–27.0) (n = 20)	0.735
Femoral rotation (°) **	- 2.1 ± 12.4 (95% CI: -7.9–3.6) (n = 20)	10.5 ± 13.4 (95% CI: 4.2–16.8) (n = 20)	0.006

Location of the first and second penetrating branches of the DFA

**Figure 3:**
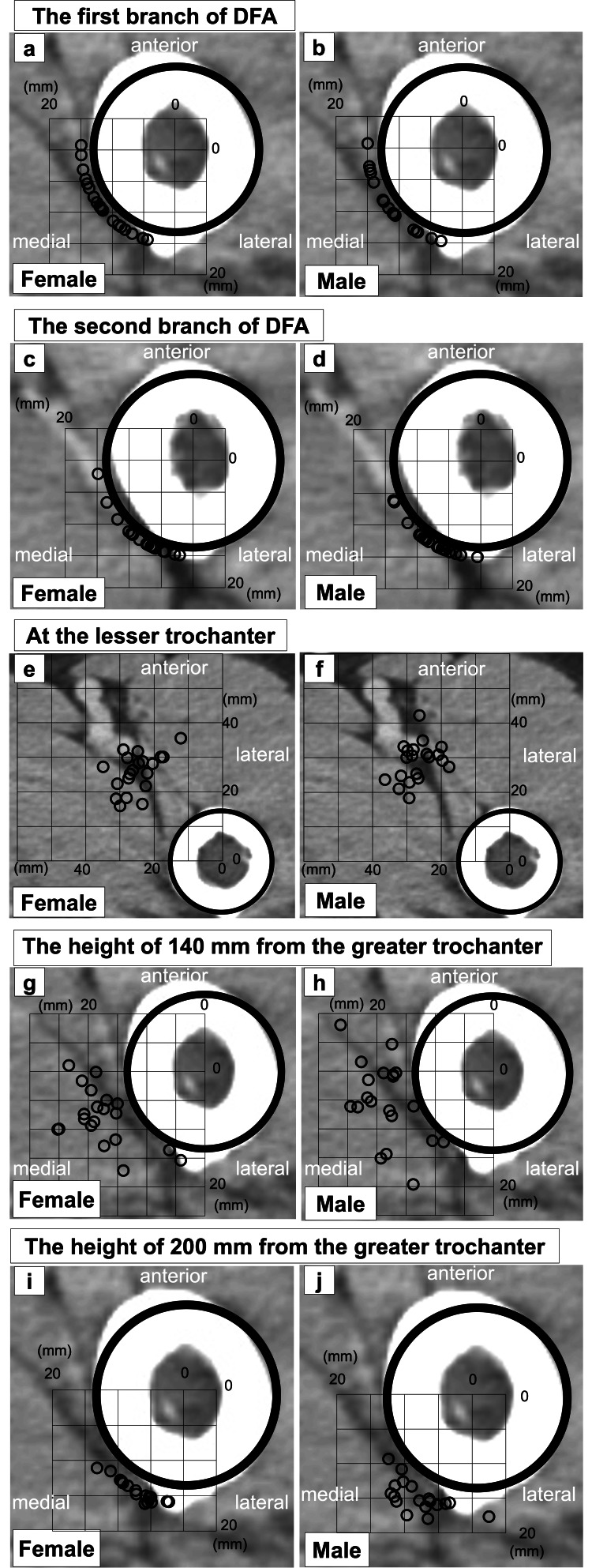
Distribution map of the deep femoral artery (DFA). Circles indicate the locations of the DFA on axial computed tomography images, reflecting positional variance among participants.

The vertical distance from the apex of the GTR to the first DFA branch was significantly longer in males than in females (113 ± 9.5 vs. 100 ± 11.7 mm, P < 0.001; Table 2). The first perforating DFA branch was identified in all participants (Fig. 3a, 3b), whereas the second branch could not be traced in one female (Fig. 3c, 3d). The height of the second DFA branch did not differ significantly between the two groups (P = 0.736). No significant sex-based differences were found in the femoral perforation angle of the first or second branches relative to the PFCA.

**Table 2:** Comparison of the perforation site, distance, and angle of the first and second perforating branches of the deep femoral artery (DFA) between the female and male participants. Data are presented as mean ± standard deviation. GTR, greater trochanter; CI, confidence interval; PFCA, posterior femoral condylar axis *Wilcoxon signed-rank test. **Angle measured with PFCA as the reference plane; positive values indicate posteromedial direction. ***The second DFA branch could not be identified on computed tomography images in one female participant.

	Female (n = 20)	Male (n = 20)	P-value*
Age (years)	65.4 ± 11.0 (95% CI: 60.2–70.6) (n = 20)	67.2 ± 10.3 (95% CI: 62.3–72.0) (n = 20)	0.481
Height (m)	1.54 ± 0.30 (95% CI: 1.49–1.56) (n = 20)	1.67 ± 0.04 (95% CI: 1.66–1.69) (n = 20)	<0.001
BMI (kg/m^2^)	24.6 ± 4.44 (95% CI: 22.5–26.7) (n = 20)	25.3 ± 3.72 (95% CI: 23.5–27.0) (n = 20)	0.735
Femoral rotation (°) **	- 2.1 ± 12.4 (95% CI: -7.9–3.6) (n = 20)	10.5 ± 13.4 (95% CI: 4.2–16.8) (n = 20)	0.006

Distance and direction of the DFA from the femur

The mean vertical distance from the GTR to the LTR was significantly greater in males than in females (81.8 ± 4.2 vs. 71.6 ± 4.9 mm, P < 0.001; Table 3). At the LTR level, the shortest DFA-femur distance in males was significantly longer than that in females (24.9 ± 4.2 vs. 21.5 ± 3.7 mm, P = 0.013). The shortest measured distance was 13.7 mm in one female. No significant differences were observed in PFCA-DFA axis angles between sexes. All DFAs were located medial to the femur (Fig. 3e, 3f).

**Table 3:** Comparison of the shortest distance from the top of the greater trochanter (GTR) to the lesser trochanter (LTR) and from the femur to the deep femoral artery (DFA) in axial images, the angle at LTR, and the shortest distances of the penetrating branches of DFA from the femur at the heights of 140 and 200 mm from the top of the GTR between males and females. Data are presented as mean ± standard deviation. CI, confidence interval *Wilcoxon signed-rank test. **Angle measured with the posterior femoral condyle axis as the reference plane; positive values indicate the anteromedial direction.

		Female	Male	P value (CI: 0.95) *
LTR	Distance from the GTR to LTR (mm)	71.6 ± 4.9 (95% CI: 69.3–73.4; range: 64–80) (n = 20)	81.8 ± 4.2 (95% CI: 79.8–83.8; range: 76–90) (n = 20)	<0.001
	Shortest distance from the femur (mm)	21.5 ± 3.7 (95% CI: 19.8–23.3; range: 13.7–29.3) (n = 20)	24.9 ± 4.2 (95% CI: 22.9–26.8; range: 17.6–34.7) (n = 20)	0.013
	Angle (°) **	46 ± 10.8 (95% CI: 41–51; range: 28–71) (n = 20)	47 ± 8.7 (95% CI: 43–51; range: 32–59) (n = 20)	0.607
140 mm	Shortest distance from the femur (mm)	5.3 ± 3.3 (95% CI: 3.8–6.9; range: 1.0–12.1) (n = 19)	5.5 ± 3.7 (95% CI: 3.7–7.2; range: 1.0–12.4) (n = 20)	0.871
	Angle (°) **	26 ± 19.5 (95% CI: 17–35; range: -2 to 75) (n = 19)	17 ± 21.4 (95% CI: 7–27; range: -17 to 55) (n = 20)	0.1261
200 mm	Shortest distance from the femur (mm)	1.3 ± 0.4 (95% CI: 1.1–1.5; range: 1.0–2.2) (n = 17)	2.2 ± 1.4 (95% CI: 1.5–2.9; range: 1.0–4.9) (n = 20)	0.017
	Angle (°) **	64 ± 11.7 (95% CI: 58–70; range: 40–82) (n = 17)	62 ± 14.4 (95% CI: 55–69; range: 37–98) (n = 20)	0.335

At 140 mm distal to the GTR, the DFA could not be traced in one female. The horizontal DFA-femur distance did not differ significantly between sexes (5.5 ± 3.7 vs. 5.3 ± 3.3 mm, P = 0.871). The shortest distance at this level was 1.0 mm. PFCA-DFA axis angles were also not significantly different (17° ± 21.4° vs. 26° ± 19.5°, P = 0.1261). In most participants, the DFA was located medial-posteriorly; however, it was positioned medial-anteriorly in four participants (one female and three males; Fig. 3g, 3h).

At 200 mm distal to the GTR, the DFA could not be traced in three females. The horizontal DFA-femur distance was significantly greater in males than in females (2.2 ± 1.4 vs. 1.3 ± 0.4 mm, P = 0.017), with the shortest measured distance being 1.0 mm. PFCA-DFA axis angles did not differ significantly (62° ± 14.4° vs. 64° ± 11.7°, P = 0.335). At this level, no DFAs were located anteriorly. Most were positioned medial-posteriorly, except for one male in whom the DFA was located lateral-posteriorly (Fig. 3i, 3j).

**Figure 4:**
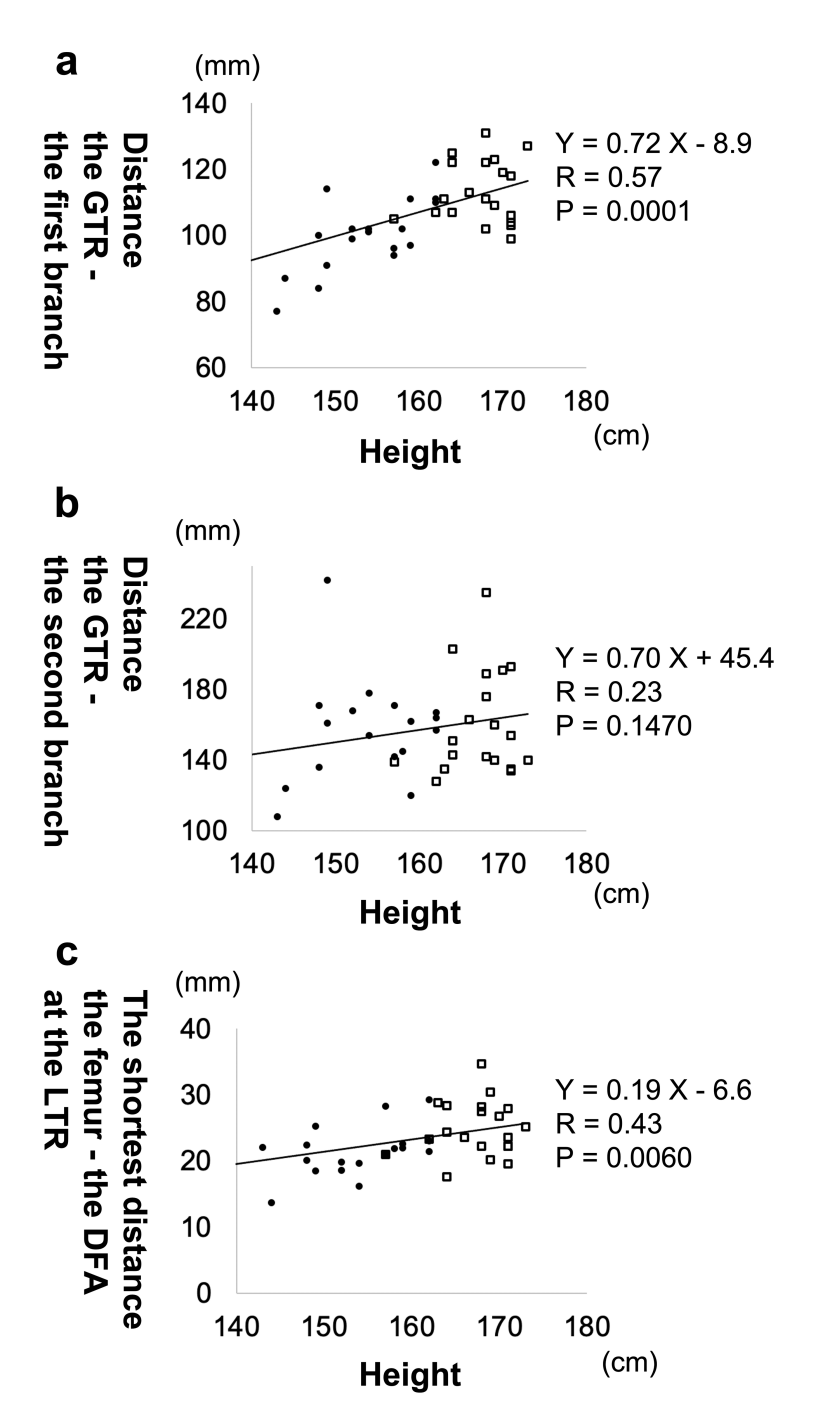
Correlation between the body height and DFA location. Graphs show the relationship between body height and the following variables: (a) distance from the GTR to the first DFA branch, (b) distance from the GTR to the second DFA branch, and (c) shortest distance from the femur to the DFA at the level of the LTR. The horizontal axis represents body height; the vertical axis represents vessel distance. Circles indicate female participants; squares indicate male participants. R, correlation coefficient; P, P-value; DFA, deep femoral artery; GTR, greater trochanter; LTR, lesser trochanter

A significant positive large correlation was observed between participant height and the vertical distance from the GTR and the first perforating DFA branch (Fig. 4; R = 0.57, P < 0.001). No significant correlation was observed between height and the distance between the GTR and the second perforating DFA branch (P = 0.147). A significant positive moderate correlation also existed between height and the shortest DFA-femur distance at the LTR level (R = 0.43, P = 0.006).

## Discussion

This study demonstrated that the height of the first perforating branch of the DFA from the apex of the GTR was shorter in females than in males. This may reflect differences in femur length associated with sex-dependent height differences [18]. Given that femur length typically correlates with body height, both the overall height and the distance from the GTR apex to the first DFA branch were positively correlated in this study. When performing wiring below the LTR for femoral trochanteric or peri-implant fractures, particular care is warranted in shorter females to avoid injury to the first perforating DFA branch. Preoperative CT imaging is recommended to assess the DFA’s course in such cases.

The mean distance from the GTR apex to the LTR was 71.6 mm in females and 81.8 mm in males. The mean height from the GTR apex to the insertion point of the first DFA branch was 100 mm in females and 113 mm in males. Thus, the average height from the LTR to the first perforating DFA branch was 28.4 mm in females and 31.2 mm in males. Cerclage wiring within this region poses a high risk of vascular injury. As the first DFA branch is thicker than other branches and supplies a substantial portion of the femur [19,20], injury can lead to serious complications, including massive bleeding. Therefore, caution is advised during cerclage wiring near this location. At the level of the LTR, the mean shortest distance between the femur and DFA was 21.5 mm in females and 24.9 mm in males. Wiring along the femur at this level can generally be performed safely within 2 cm of the femur in both sexes. However, the shortest recorded distance among all 40 participants was 13.7 mm, observed in a female aged ≥80 years with a height of 143 cm. As shown in Fig. 4c, shorter participants had a smaller distance between the DFA and femur at levels below the LTR. Therefore, greater caution is needed in shorter females, and preoperative measurement of the shortest DFA-femur distance via CT is recommended when planning cerclage wiring.

At a height of 140 mm from the GTR apex, the DFA was predominantly located medial-posteriorly. However, after correcting for femoral rotation, it was found in a medio-anterior position in four participants (10.0%; one female, three males). This poses a risk of DFA injury during closed reduction and fixation with intramedullary nailing, particularly during distal screw insertion, often performed at the anteversion angle of the femoral neck. At 200 mm from the apex, the DFA was not located medial-anteriorly in any case, suggesting this height is safer for distal screw placement. Although the overall risk of vascular injury during hip fracture surgery is low (approximately 0.2%), it may result from damage by screws or bone fragments [21]. To prevent such injuries, surgeons should avoid excessive femoral internal rotation, refrain from compressing the medial femur using bars between the hip joints on traction tables, and carefully align distal screws to avoid medial-posterior trajectories that oppose femoral neck anteversion [22]. 

The trajectory of the DFA has direct clinical relevance, particularly in orthopaedic procedures involving screw insertion into the distal femur, such as intramedullary fixation, when the course of the DFA closely aligns with the typical direction of screw placement. While our study identified significant sex differences, we also found that patient height played an important role in DFA proximity: shorter individuals tended to have a smaller distance between the femur and the DFA. This suggests that anatomical variability extends beyond sex alone.

Previous studies, such as those by Apivatthakakul et al. and Tomaszewski et al., have similarly emphasized the risks associated with DFA variability and its implications for surgical safety [1,23]. In terms of surgical scenarios, our findings may have a greater impact on procedures such as femoral nailing, where distal screw insertion at the level of femoral anteversion poses a higher risk of vascular injury. In contrast, DFA injury is less frequently reported during primary THA, but may become a concern during revision THA, particularly when cerclage wiring is used for femoral stem fixation or fracture management. In such cases, the course of the DFA along the medial aspect of the femur may place it at increased risk of iatrogenic injury. Tailoring surgical strategies to patient-specific vascular anatomy, such as utilizing preoperative CT, may help reduce complications in both trauma and revision arthroplasty settings.

This study has several limitations. First, we retrospectively analyzed only contrast-enhanced CT venous phase images from the unaffected side, which was always contralateral and free of radiographic osteoarthritis. The affected side was excluded due to frequent metal artifacts and anatomical distortion caused by osteoarthritis or postoperative changes, which made accurate evaluation of the DFA course difficult. Second, bilateral analysis was not conducted, which may limit applicability to surgical planning in patients with bilateral disease or deformity. Third, measurements of femoral height on coronal sections may have inherent inaccuracies due to femoral bowing. Fourth, the use of 1.5-mm slice thickness may limit the visualization of smaller vascular branches. Lastly, no cadaveric or intraoperative validation was performed to verify imaging findings. Future research should prospectively examine changes in the DFA before and after surgery in cases involving significant lower limb shortening, such as proximal femoral fractures or two-stage revision THA. Such studies would help understand and reduce DFA injury risks during surgery.

## Conclusions

Careful femoral wiring is important, as the first perforating branch of the DFA tends to be located more proximally and medially in females. While our findings suggest zones where caution may be needed, these should not be interpreted as definitive “safe” or “avoidance” areas without further validation. Imaging-based suggestions must be confirmed through prospective clinical studies. Patient-specific vascular anatomy should be considered when planning femoral internal fixation, especially in revision cases.
